# Inhibitory Effects of *Artemisia argyi* Extracts on *Microcystis aeruginosa*: Anti-Algal Mechanisms and Main Allelochemicals

**DOI:** 10.3390/biology14091141

**Published:** 2025-08-29

**Authors:** Jiajia Dong, Peng Li, Yalei Du, Lingling Cao, Zhiqiang Yan

**Affiliations:** 1 School of Life Sciences, Nanyang Normal University, Nanyang 473061, China; dong20231120@163.com (J.D.); pengli082021@163.com (P.L.); dyl2024315@163.com (Y.D.); 2 College of Water Resources and Modern Agriculture, Nanyang Normal University, Nanyang 473061, China; cao_ling2019@163.com; 3 Collaborative Innovation Center of Water Security for Water Source Region of Mid-Line of South-to-North Diversion Project of Henan Province, Nanyang Normal University, Nanyang 473061, China

**Keywords:** *Artemisia argyi*, allelopathy, *Microcystis aeruginosa*, photosynthesis, ROS, flavonoid

## Abstract

Harmful cyanobacterial blooms (CyanoHABs) threaten freshwater ecosystems and human health. Inhibiting cyanobacteria through plant allelopathy is an effective and environmentally friendly approach for CyanoHAB control. In this study, we evaluated the inhibitory activities of several organic solvent extracts from *Artemisia argyi* against the common bloom-forming cyanobacterium *Microcystis aeruginosa*, explored the anti-algal mechanism of the active fraction, analyzed its secondary metabolites using liquid chromatography–high-resolution mass spectrometry (LC-HRMS), and screened the potential allelochemicals. The results showed that the crude extract of *A. argyi* leaves (CE) exhibited significant inhibitory effects on *M. aeruginosa*. Among several solvent fractions of CE, the dichloromethane extract (DE) demonstrated the strongest inhibitory effect, with a 7-day IC_50_ of 70.43 mg/L. After treatment with DE, the contents of chlorophyll a (Chl a), carotenoids, and phycobiliproteins (PBPs) in *M. aeruginosa* were significantly reduced. Meanwhile, an excessive accumulation of reactive oxygen species (ROS), reduction of catalase (CAT) activity, increase in malondialdehyde (MDA) content, and shrinkage of the membrane were found in *M. aeruginosa* cells under DE treatments. There were 81 secondary metabolites annotated in DE by LC-HRMS. Among them, hispidulin, jaceosidin, 5,7,3′-trihydroxy-6,4′,5′-trimethoxyflavone, and eupatilin possessed strong inhibitory activities, with 7-day IC_50_ values of 26.23, 27.62, 32.02, and 34.98 mg/L, respectively. These results indicated that the *A. argyi* extracts possess significant allelopathic activities on *M. aeruginosa*, and DE was identified as the primary active fraction. It inhibits algae growth by suppressing photosynthesis and inducing peroxidation, ultimately leading to cell death. Flavonoids in DE were the main allelochemicals responsible for the inhibition on algae of *A. argyi* extracts.

## Simple Summary

Harmful cyanobacterial blooms caused by *Microcystis aeruginosa* threaten aquatic ecosystems. This study explores the inhibitory activities and anti-algal mechanisms of *Artemisia argyi* extracts against *M. aeruginosa* and identifies the allelochemicals. The dichloromethane extract of *A. argyi* leaves (DE) exhibited the strongest inhibitory effects on *M. aeruginosa*. DE could suppress the photosynthesis and induce excessive accumulation of ROS in *M. aeruginosa* cells. Flavonoids were the main secondary metabolites in DE and possessed strong anti-algal activities. These findings highlight the potential of using *A. argyi* extracts as an effective and environmentally friendly strategy for controlling harmful algal blooms.

## 1. Introduction

Under the influence of population growth, economic development, and climate change, eutrophication of water is becoming increasingly severe worldwide. Harmful cyanobacterial blooms (CyanoHABs), resulting from water eutrophication, pose a significant challenge to freshwater ecosystems [1]. Traditional bloom control methods, such as physical removal and the application of chemical algaecides, are often costly and can cause secondary pollution to aquatic environments [2,3]. In recent years, the utilization of allelochemicals released by plants to inhibit algal growth has gained considerable attention [4].

Many aquatic and terrestrial plants can release allelochemicals that inhibit the growth of bloom-forming algae and prevent the occurrence of blooms. The activity of allelochemicals exhibits high specificity, and they are readily degradable under natural conditions with no or little ecological harm [4,5]. Macrophytes can suppress phytoplankton growth by releasing allelochemicals, showing the application value to control CyanoHABs [6]. Consequently, plant-derived allelochemicals represent an environmentally friendly approach for controlling algal growth.

The approaches of inhibiting algae by plant allelopathy include planting macrophytes and the direct addition of extracted allelochemicals. For instance, Maxine et al. carried out an ex situ experiment with multiple planting densities of six emergent macrophyte species on the phytoplankton community of a tropical reservoir, and the results showed that *Ludwigia adscendens* and *Persicaria barbata* significantly reduced the relative biovolume of cyanobacteria in the phytoplankton communities, indicating emergent macrophyte species could reduce total phytoplankton biomass and cyanobacterial dominance in tropical water bodies [7]. *Ceratophyllum demersum* could release more allelochemicals in coexistence with toxic strains of *M. aeruginosa* under the presence of microcystins (MCs), displaying potential to control toxic and non-toxic cyanobacterial blooms [8]. The extracts of *Salix atrocinerea* suppressed the phototrophic growth of *Planktothrix agardhii* (a cyanobacterial) at very low concentrations but had no effect on the growth of *Scenedesmus communis* (a chlorophyte), indicating that *S. atrocinerea* extract could act as a promising agent selectively affecting cyanobacterial growth [9]. *Carex cinerascens* extracts of concentrations more than 1.5 mg/L inhibited the colony formation in *M. aeruginosa*, contributing to the control of bloom formation [10].

Up to now, a lot of allelochemicals exhibiting inhibitory effects on freshwater algae have been identified from plants, including polyphenolics, N-containing compounds (e.g., alkaloids), fatty acids/esters, terpenoids, and their derivatives [4]. For example, organic acids from *Portulaca oleracea* [11], triterpenes from *Lantana camara* [12], and chiral flavonolignans from barley straw (*Hordeum vulgare*) [13] exhibited significant inhibitory effects against bloom-forming algae.

Recently, research on the mechanism of action by which allelochemicals inhibit the growth of algae has been advancing progressively deeper. Allelochemicals induce damage at multiple levels of microalgal cells, including interfering with photosynthesis, generating oxidative stress, triggering programmed cell death (PCD), and disturbing other physiological and biochemical processes [4]. The allelochemical *p*-coumaric acid significantly inhibited the growth of the bloom-forming cyanobacteria *Limnothrix* sp. in a dose-dependent manner. The allelochemical can lead to the destruction of algae cell structure, leakage of intracellular material, reduced photosynthetic pigments and protein content, inhibited photosynthesis, and stress on the antioxidant system [14]. Juglone depressed the cell proliferation of *M. aeruginosa* by inducing oxidant stress in algal cells [15]. Tannic acid effectively inhibited *M. aeruginosa* growth and microcystin-leucine-arginine (MC-LR) production. Under tannic acid exposure, proteins related to the tricarboxylic acid (TCA) cycle, glycolysis, and leucine and arginine biosynthesis were upregulated, while proteins involved in ion and metal-cluster binding, disrupting electron transfer, and photosynthesis were downregulated [16].

*A. argyi*, a perennial plant species belonging to the genus *Artemisia* (Asteraceae family), is widely distributed in middle and low elevations across Asia, Europe, and the Americas. Its leaves constitute the plant’s primary medicinal part [17]. Previous studies showed that *A. argyi* had allelopathic effects on target plants. In pot experiments, *A. argyi* could inhibit seed germination and seedling growth of *Brassica pekinensis*, *Lactuca sativa*, *Oryza sativa*, *Portulaca oleracea*, *Oxalis corniculata*, and *Setaria viridis*, displaying strong allelopathic effects [18]. The aqueous extracts of *A. argyi* leaves displayed strong inhibitory effects on rice growth, and the allelopathic action was related to the hormone balance, element absorption, and photosynthesis of receptor plants [19]. There are a lot of secondary metabolites in *A. argyi*, such as sesquiterpenoids, triterpenoids, monoterpenoids, flavonoids, fatty acids, organic acids, essential oils, etc. [20,21,22]. Chen et al. isolated and identified 14 compounds from the aqueous extract of *A. argyi* leaves, including 13 phenolic compounds. They found that caffeic acid and isochlorogenic acid A exhibited significant inhibitory effects on several weed species [23,24]. In addition to exhibiting allelopathic effects on the seed germination and seedling growth of weeds and crops, the aqueous extract of the whole *A. argyi* plant also demonstrated significant inhibitory effects against *M. aeruginosa* [25]. However, investigations of the allelopathic effects of organic solvent extracts from *A. argyi* leaves on algae have not been reported.

In the present study, *M. aeruginosa*, the common algae species in CyanoHABs and the most sensitive microalgal species to allelochemicals, was used as the target algae. The aim of this study was to (1) evaluate the concentration effects of *A. argyi* crude extract and extracts with several organic solvents of different polarities on *M. aeruginosa*; (2) explore the mechanism of action of DE on *M. aeruginosa*; (3) analyze the compounds in DE through LC-HRMS; and (4) verify the potential allelochemicals that played roles in the inhibitory effects of *A. argyi* on *M. aeruginosa*. This study might provide some more knowledge about the allelopathic effects of *A. argyi* on an algae and the potential active compounds that act in the process. To the best of our knowledge, this is the first report about the allelopathic effects of organic solvent extracts of *A. argyi* on micro-algae and the identification of anti-algal allelochemicals.

## 2. Materials and Methods

### 2.1. Algal Culture

*Microcystis aeruginosa* (strain FACHB-977) was obtained from the Freshwater Algae Culture Collection at the Institute of Hydrobiology, Chinese Academy of Sciences. The algae was cultured in BG11 medium under the following conditions: light intensity of 25 μmol photons m^−2^ s^−1^, temperature of 25 ± 1 °C, and a light/dark cycle of 12 h:12 h. All experiments were conducted using algae in the exponential growth phase.

### 2.2. Extracts of A. argyi, Compounds

The fresh leaves (17.13 kg) of *A. argyi* were harvested from the cultivation base of Nanyang Academy of Agricultural Sciences in Henan Province on 6 June 2022. After drying at room temperature, the leaves (3.64 kg) were pulverized into a fine powder and extracted with 95% ethanol (5 L × 3) at room temperature. The extract was then concentrated using a rotary evaporator (IKA RV3 eco, IKA Works GmbH & Co., Staufenim Breisgau, Germany) to obtain the crude extract of A. argyi leaves (CE, 0.82 kg). CE was then partitioned successively with petroleum ether (60–90 °C, 500 mL × 3), dichloromethane (500 mL × 3) and ethyl acetate (500 mL × 3). After concentration and solvent removal, the extracts of petroleum ether (PEE), dichloromethane (DE), ethyl acetate (EE), and the aqueous phase (WP) were obtained and stored at 4 °C before use.

The flavonoids (Eupatilin, 5,7,3′-Trihydroxy-6,4′,5′-trimethoxyflavone, Jaceosidin, Chrysosplenetin B, Hispidulin, and Isofraxidin) and coumarins (Scopoletin and 7-Hydroxycoumarin) of HPLC grade were acquired from Shanghai Macklin Biochemical Technology Co., Ltd. (Shanghai, China).

### 2.3. Anti-Algal Bioassay

Extracts of *A. argyi* leaves were dissolved in DMSO (99.9%, Macklin Biochemical Co., Ltd., Shanghai, China) to prepare stock solutions at concentrations of 50, 100, and 200 g/L. The stock solutions were added to algal suspensions with final concentrations of 50, 100, and 200 mg/L, respectively. The initial cell density of *M. aeruginosa* was (3.17 ± 0.23) × 10^6^ cells/mL in the system. Flavonoids and coumarins were dissolved in DMSO to prepare a stock solution, which was added to the algal suspensions at a ratio of 0.1% (*v*/*v*). The control group was supplemented with an equal ratio of DMSO. All experiments were performed in triplicate and maintained for 7 days. The OD_680_ of algal solutions was determined every 24 h. The algal cell density was calculated based on the OD_680_ using the formula:

Y = 11.981X + 0.2078, R^2^ = 0.9914 (1)

Y, cell density, cells/mL.

X, OD_680_.

The inhibition rate (IR) was calculated using the formula:

IR (%) = (1 − E/C) × 100%(2)

E, cell density of treated groups, cells/mL.

C, cell density of control group, cells/mL.

### 2.4. Pigment Contents

According to the IC_50_ value of DE against *M. aeruginosa*, treatment groups were established at concentrations corresponding to 0.5 × IC_50_, 1 × IC_50_, and 2 × IC_50_, achieving final DE concentrations of 35, 70, and 140 mg/L, respectively. Cell samples were collected daily from days 1 to 7, in 10 mL volumes. These were then centrifuged at 8000 *g* for 5 min at 4 °C. The supernatant was discarded, and 4 mL of 95% ethanol was added to resuspend the algal cells. The suspension was then extracted in the dark at 4 °C for 24 h. Subsequently, the samples were centrifuged at 8000× *g* for 10 min at 4 °C. The supernatant was collected, and its absorbance was measured at wavelengths of 665, 649, and 470 nm. The Chl a and carotenoid contents were determined according to the following formulas:

C_Chl a_ = 13.95 × OD_665_ − 6.88 × OD_649_ (3)

C_Carotenoid_ = (1000 × OD_470_ − 2.05 × Chl a) ÷ 245 (4)

C_Chl a_, concentration of Chl a, mg/L.

C_Carotenoid_, concentration of carotenoid, mg/L.

The suspension was subjected to repeated freeze-thaw cycles, followed by centrifugation at 3000 g for 10 min, 4 °C. The supernatant was collected, and its absorbance was measured at 650, 620, and 565 nm. The concentrations of phycocyanin (PC), allophycocyanin (APC), and phycoerythrin (PE) were calculated according to the following formulas [26]:

PC = (OD_620_ − 0.7 × OD_650_) ÷ 7.38 (5)

APC = (OD_650_ − 0.19 × OD_620_) ÷ 5.65 (6)

PE =(OD_565_ − 2.8 × PC − 1.34 × APC) ÷ 1.27 (7)

The PBP contents of *M. aeruginosa* were expressed in μg/10^6^ cells.

### 2.5. Determination of pH and EC, Morphological Characterization

#### 2.5.1. Measurements of pH and EC

A 5 mL sample of the algal culture was taken daily, and the pH was measured using a pH meter (FE28-CN, METTLER TOLEDO, Zurich, Switzerland). For electrical conductivity (EC), the algal culture of 5 mL was centrifuged at 8000 rpm for 15 min at 4 °C. The supernatant was then transferred to a new centrifuge tube, and the EC was measured using a conductivity meter (FE32 -Standard, METTLER TOLEDO, Zurich, Switzerland).

#### 2.5.2. SEM

The morphological characteristics of *M. aeruginosa* were examined using scanning electron microscopy (SEM, GeminiSEM 300, ZEISS, Jena, Thüringen, Germany). An algal suspension sample of 50 mL was centrifuged at 7000 rpm for 10 min, and the supernatant was discarded. Then, 2 mL of pre-chilled (4 °C) 2.5% glutaraldehyde fixative was slowly added along the tube wall, and the samples were fixed overnight at 4 °C. After centrifugation to remove the fixative, the samples were washed three times with phosphate buffer solution (0.1 M, pH 7.0). Subsequently, the samples were dehydrated using an ethanol gradient series (30%, 50%, 70%, 80%, 90%, and 95%), with each step lasting 15 min. This was followed by two rinses with absolute ethanol (20 min each). Next, the samples were treated with a mixture of absolute ethanol and isoamyl acetate (*V*/*V* = 1:1) for 30 min and then fully immersed in pure isoamyl acetate overnight. After centrifugation to remove the supernatant, the concentrated algal cells were freeze-dried in a lyophilizer for 12 h. Finally, the samples were gold-coated and examined under a scanning electron microscope to observe the ultrastructural changes in *M. aeruginosa* cells.

### 2.6. Determination of Oxidative Stress Levels and Antioxidant Enzyme Activities

#### 2.6.1. Sample Preparation

On days 1, 3, 5, and 7 of the experiment, 50 mL of algal culture was collected and centrifuged at 4 °C and 8000 rpm for 5 min to concentrate. The pellet was resuspended in 1 mL of PBS and subjected to ultrasonic disruption for 5 min using a cell disruptor (300 W, 5 s on/30 s off) in an ice bath. The disrupted algal cells were then centrifuged at 4 °C and 12,000 rpm for 15 min. The resulting supernatant (crude enzyme extract) was stored at −20 °C for subsequent assays.

#### 2.6.2. TP

Protein molecules contain -NH_3_^+^ groups. When Coomassie Brilliant Blue (CBB) dye is added to protein standards or samples, the anionic dye binds to -NH_3_^+^, turning the solution blue. The protein content is calculated by measuring the absorbance. Reagent preparation and procedures followed the Nanjing Jiancheng Kit protocol.

(8) $$Protein\;concentration(g/L)=\frac{A_{sample}-A_{blank}}{A_{standard}-A_{blank}}\times C_{standard}\times N$$

N: Sample dilution factor before testing.

#### 2.6.3. MDA

The thiobarbituric acid (TBA) method was used. MDA, a product of lipid peroxidation, reacts with TBA to form a red adduct with maximum absorbance at 532 nm. Reagent preparation and procedures followed the Nanjing Jiancheng Kit protocol.

(9) $$\mathrm{MDA}\;\mathrm{concentration}\left(\frac{nmol}{mg}prot\right)=\frac{{\mathrm{OD}}_{sample}-{\mathrm{OD}}_{blank}}{{\mathrm{OD}}_{standard}{-\mathrm{OD}}_{blank}}\times standard\;concentration\left(\frac{10\;nmol}{1\;mL}\right)\;÷sample\;protein\;concentration(\frac{mg\;prot}{mL})$$

#### 2.6.4. H_2_O_2_

H_2_O_2_ reacts with molybdate to form a complex, and its concentration is determined by measuring the absorbance at 405 nm. Reagent preparation and procedures followed the Nanjing Jiancheng Kit protocol.

(10) $$H_{2}O_{2}\;content\left(\frac{mmol}{g\;prot}\right)=\frac{A_{sample}-A_{blank}}{A_{standard}-A_{blank}}\times C_{standard}÷C_{pr}$$

C_pr_: Tissue homogenate protein concentration (g prot/L, “prot” denotes protein).

#### 2.6.5. CAT Activity

Ammonium molybdate stops the CAT reaction and forms a light-yellow complex with residual H_2_O_2_. CAT activity is calculated based on the change in H_2_O_2_ concentration. Reagent preparation and procedures followed the Nanjing Jiancheng Kit protocol.

(11) $$CAT\;activity(U/mgprot)=∆A\times 271÷V_{sample}÷T÷C_{pr}$$

V_sample_: Sample volume (0.1 mL).

T: Reaction time (60 s).

C_pr_: Homogenate protein concentration (mg prot/mL).

### 2.7. LC-HRMS

HPLC analysis was performed on an ACQUITY UPLC HSS T3 column (2.1 mm × 100 mm, 1.8 μm, Waters, Milford, CA, USA). The mobile phase consisted of two solvents: solvent—(A) deionized water with 0.1% formic acid, and solvent (B)—acetonitrile with 0.1% formic acid.

Gradient elution was performed at a flow rate of 0.3 mL/min at room temperature. The elution profile was isocratic from 0 to 10 min (100% (A), 0% (B)), from 10 to 25 min (70% (A), 30% (B)), from 25 to 30 min (60% (A), 40% (B)), from 30 to 40 min, isocratic (50% (A), 50% (B)), from 40 to 45 min (30% (A), 70% (B)), from 45 to 60 min (0% (A), 100% (B)), and from 60 to 70 min (100% (A), 0% (B)).

The high-resolution mass spectrometry analysis was performed using a Q-Exactive Orbitrap (Thermo Fisher Scientific, San Jose, CA, USA) equipped with a heated electrospray ionization (HESI) source. Detection was carried out in Full MS-ddMS^2^ mode, with separate scans in positive and negative ion modes. The scan range was set to *m*/*z* 100–1200. The MS^1^ resolution was set to 70,000, and the MS^2^ resolution was set to 17,500. The ion source voltage was 3.2 kV. The capillary temperature was maintained at 320 °C, and the auxiliary gas heater temperature was set to 350 °C. The sheath gas flow rate was 40 L/min, and the auxiliary gas flow rate was 15 L/min. The automatic gain control (AGC) target was set to 1 × 10^6^, and the TopN setting was 5. MS^2^ scans were triggered using stepped normalized collision energy (NCE) set to 30, 40, and 50.

Data were processed by Compound Discover 3.2 (Thermo Fisher Scientific, CA, USA) and compared with databases including PubChem (https://pubchem.ncbi.nlm.nih.gov/, accessed on 17 December 2022), ChEBI (https://www.ebi.ac.uk/chebi/init.do, accessed on 17 December 2022), ChEMBL (https://www.ebi.ac.uk/chembl/, accessed on 17 December 2022), and mzCloud (https://www.mzcloud.org/, accessed on 17 December 2022) to annotate compounds. All compounds have been assigned based on level 3 annotation, which refers to the assignment of metabolites based on spectral data [27].

### 2.8. Statistical Analyses

Data were processed and plotted with Excel 2013 and Origin 2021. Statistical analysis was conducted with IBM SPSS 26. One-way analysis of variance (ANOVA) followed by the Tukey test was applied to assess the statistical significance of differences among different groups. The differences were identified as significant at *p* < 0.05. The half-maximal inhibitory concentration (IC_50_) for *A. argyi* extracts and compounds against *M. aeruginosa* was derived using a linear fitting curve that correlated the treatment concentration with corresponding IR values in GraphPad Prism software (version 9.5.1).

## 3. Results

### 3.1. Effects of Extracts of A. argyi Leaves on M. aeruginosa

**Figure 1:**
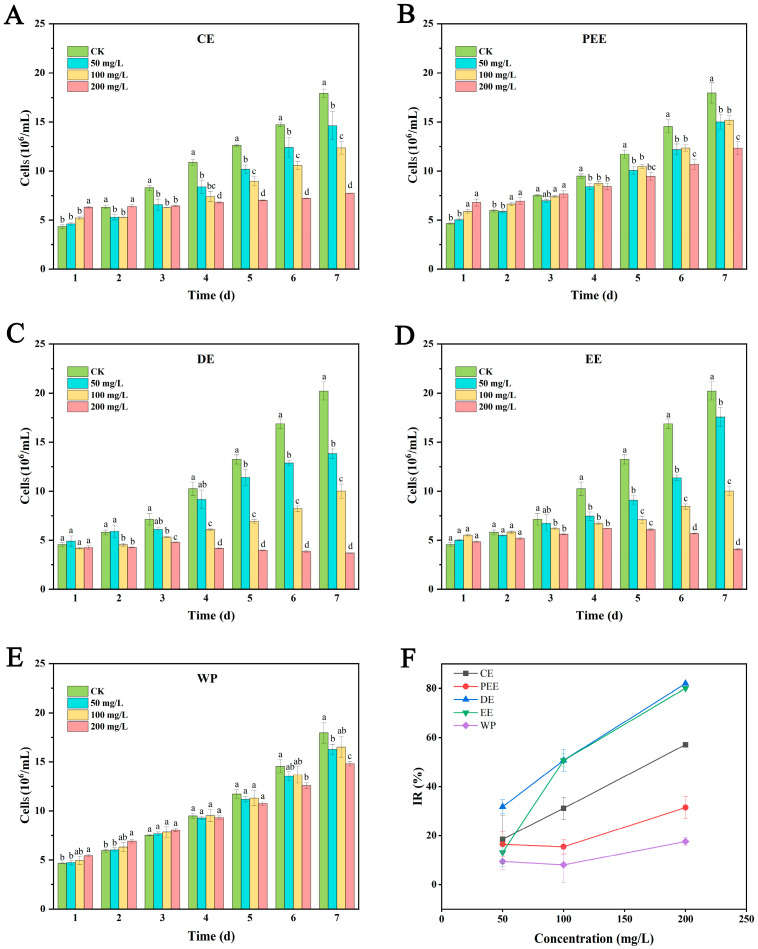
Cell densities of *M. aeruginosa* after treatments with CE (**A**), PEE (**B**), DE (**C**), EE (**D**), and WP (**E**), and the IRs (**F**) of the extracts on day 7. The results presented are the mean of three replicates ± SE; different letters denote significant differences at *p* < 0.05 according to one-way ANOVA with an LSD test.

From day 3, the growth of *M. aeruginosa* cells exposed to CE began to be inhibited. The cell densities at all treated concentrations were significantly lower than that of control (Figure 1A). The cell densities of *M. aeruginosa* under all concentrations of PEE were significantly lower than those in the control from days 4 to 7 (Figure 1B). DE exhibited a concentration-dependent inhibitory effect on *M. aeruginosa* cell growth. From day 2, the algal cell densities under treatment with DE of 100 and 200 mg/L were significantly lower than those in the control (Figure 1C). EE at concentrations of 100 and 200 mg/L demonstrated significant inhibitory activities on algal cell growth from day 3 to 7 (Figure 1D). WP showed weak inhibitory activity on *M. aeruginosa* cell growth, with significantly lower algal cell densities observed only at 200 mg/L on days 6 and 7 compared to the control (Figure 1E). On day 7, the IRs of CE, PEE, DE, EE, and WP on *M. aeruginosa* growth were 57.09%, 31.44%, 82.01%, 80.06%, and 17.61%, respectively (Figure 1F). The IC_50_ values for CE, DE, and EE on algal cell growth on day 7 were 179.54, 70.43, and 81.29 mg/L, respectively. DE displayed the strongest anti-algal effect of all extracts of *A. argyi* leaves.

### 3.2. Influence of DE on Photosynthetic Pigment Contents in M. aeruginosa Cells

**Figure 2:**
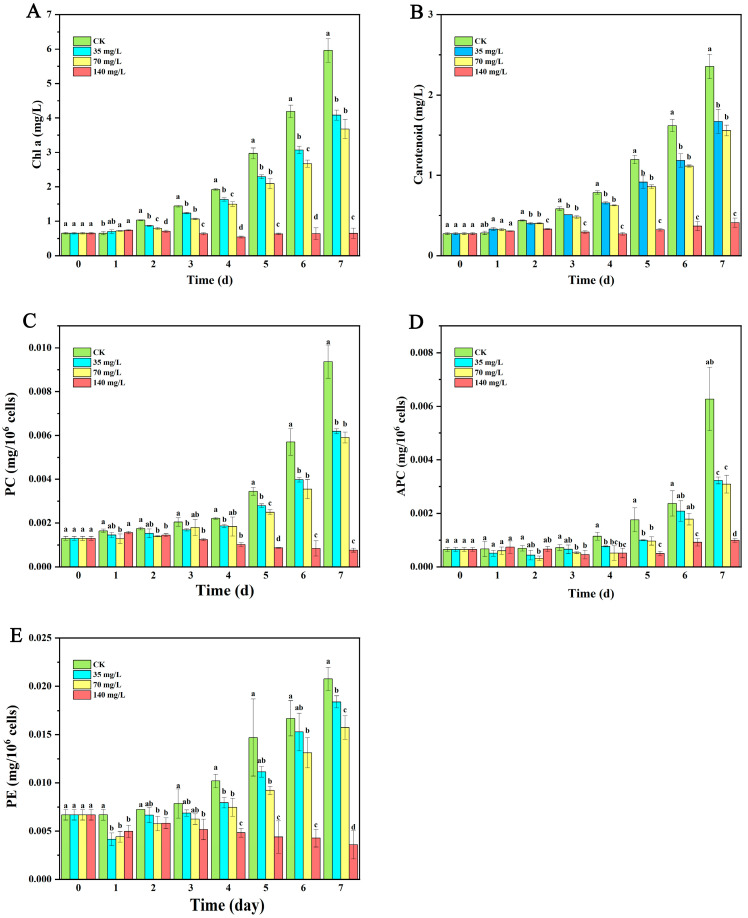
Chl a (**A**), carotenoids (**B**), PC (**C**), APC (**D**), and PE (**E**) contents in *M. aeruginosa* cells after treatments with DE. The results presented are the mean of three replicates ± SE; different letters denote significant differences at *p* < 0.05 according to one-way ANOVA with an LSD test.

From days 2 to 7, the Chl a content in *M. aeruginosa* cells treated with DE at all concentrations was significantly lower than that of the control group. By day 7, the Chl a content under 140 mg/L DE was 0.648 mg/L, representing only 10.87% of the control value (Figure 2A). Starting from day 2, DE at all concentrations significantly inhibited the carotenoid content in *M. aeruginosa* cells, with a more pronounced decrease observed over time. Particularly on day 7, the carotenoid content in algal cells treated with 140 mg/L DE was 0.41 mg/L, amounting to only 17.4% of the control (Figure 2B). DE significantly suppressed the phycobiliprotein (PBP) content in *M. aeruginosa*. From days 5 to 7, the phycocyanin (PC) content in all treatment groups was significantly lower than that of the control (Figure 2C). The effect of DE on the allophycocyanin (APC) content was concentration-dependent: DE at 35, 70, and 140 mg/L significantly inhibited the APC content starting from days 4, 3, and 2, respectively (Figure 2D). Following DE treatment, the phycoerythrin (PE) content exhibited a decreasing trend with prolonged exposure time and increasing concentration (Figure 2E). On day 7, after treatment with 140 mg/L DE, the PC, APC, and PE contents in algal cells were 8.15%, 15.75%, and 17.22% of the control values, respectively.

### 3.3. Oxidative Stress of DE on M. aeruginosa

**Figure 3:**
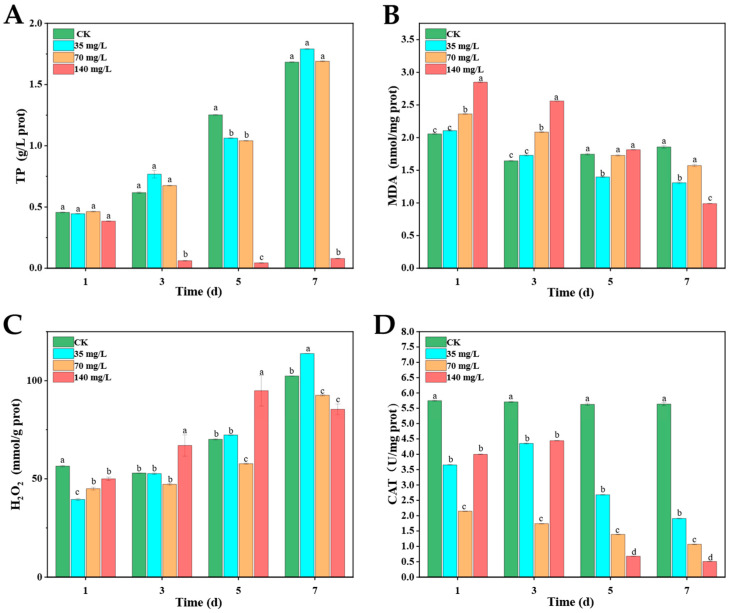
TP (**A**), MDA (**B**), and H_2_O_2_ (**C**) levels and CAT activities (**D**) in *M. aeruginosa* cells after treatments with DE. The results presented are the mean of three replicates ± SE; different letters denote significant differences at *p* < 0.05 according to one-way ANOVA with an LSD test.

As shown in Figure 3A, the total protein (TP) content of *M*. *aeruginosa* continuously increased in CK, reflecting normal growth and protein synthesis. Under 35 and 70 mg/L DE, the TP levels were similar to that of the control, while the TP contents were significantly decreased by treatment with DE at 140 mg/L. After treatments with DE, the malondialdehyde (MDA) content in *M*. *aeruginosa* cells initially increased at days 1 and 3 but gradually decreased at days 5 and 7 (Figure 3B). Under 140 mg/L DE, the H_2_O_2_ levels in *M*. *aeruginosa* cells were significantly higher than those of CK at days 3 and 5, while the H_2_O_2_ levels under 35 and 70 mg/L DE were similar to those of CK (Figure 3C). The CAT activities were strongly inhibited by DE at all treated concentrations from days 1 to 7. Especially on day 7 under 140 mg/L, the CAT activity was only 11.36% of CK (Figure 3D).

### 3.4. Effects of DE on Cell Permeability of M. aeruginosa

**Figure 4:**
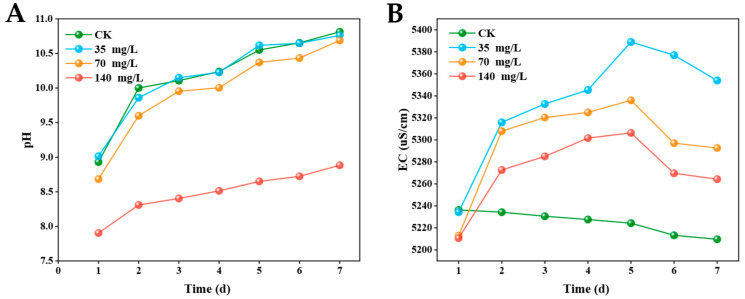
The pH (**A**) and EC (**B**) in *M. aeruginosa* cultures after treatments with DE.

**Figure 5:**
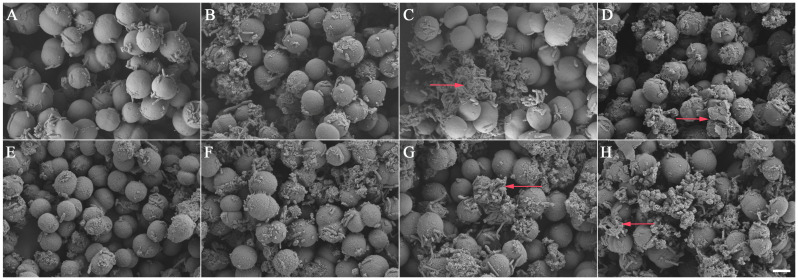
SEM of cell surface structures of *M. aeruginosa* after treatments with DE. Panels (**A**–**D**) represent day 3 of the experiment, where (**A**) is the control group, and (**B**–**D**) correspond to 35, 70, and 140 mg/L treatment groups, respectively. Panels (**E**–**H**) represent day 7, with (**E**) as the control and (**F**–**H**) showing 35, 70, and 140 mg/L treatments. The red arrows indicate membrane damages of *M. aeruginosa* cells. Scale bar = 2 μm.

Under 35 and 70 mg/L DE, the pH of *M. aeruginosa* cultures was similar to that of CK. After treatments with DE of 140 mg/L, the pH values were significantly lower than those with CK (Figure 4A). The EC changes in *M. aeruginosa* cultures following treatment with DE are shown in Figure 4B. In CK, the EC values fluctuated within the range of 5236–5250 μS/cm without a distinct trend. For the treatment groups, the EC values exhibited a gradual increase from day 2. Scanning electron microscopy (SEM) was employed to analyze the morphology of *M. aeruginosa* cells (Figure 5). Cells in the control groups exhibited turgid morphology with smooth, rounded surfaces at days 3 and 7 (Figure 5A,E). After 3 days of treatments with DE at 35 and 70 mg/L, the *M. aeruginosa* cells showed initial deformation and surface depression, and advanced cellular collapse with membrane rupture was evident under 140 mg/L (Figure 5B–D). When the exposure time was prolonged to 7 days, the *M. aeruginosa* cells developed rough surfaces with mucous filaments, membrane damage, and cellular fusion under DE of 35 and 70 mg/L, and complete loss of normal morphology with cellular collapse was found under 140 mg/L (Figure 5F–H).

### 3.5. LC-HRMS Analysis of Secondary Metabolites in DE

LC-HRMS analysis of DE annotated 81 secondary metabolites (Table 1 and Table S1), including flavonoids, coumarins, phenolic acids, terpenoids, alkaloids, fatty acids, and others. Flavonoids and coumarins were present at relatively higher levels. Among these, six flavonoids exhibited the following relative abundances: eupatilin (25.187%), jaceosidin (14.614%), 5,7,3′-trihydroxy-6,4′,5′-trimethoxyflavone (6.754%), hispidulin (4.377%), chrysosplenetin B (8.442%), and isofraxidin (2.342%). The relative abundances of two coumarins, scopoletin and 7-hydroxycoumarin, were 5.537% and 3.474%, respectively (Table 1).

### 3.6. The Inhibitory Effects of Main Flavonoids in DE on M. aeruginosa

**Figure 6:**
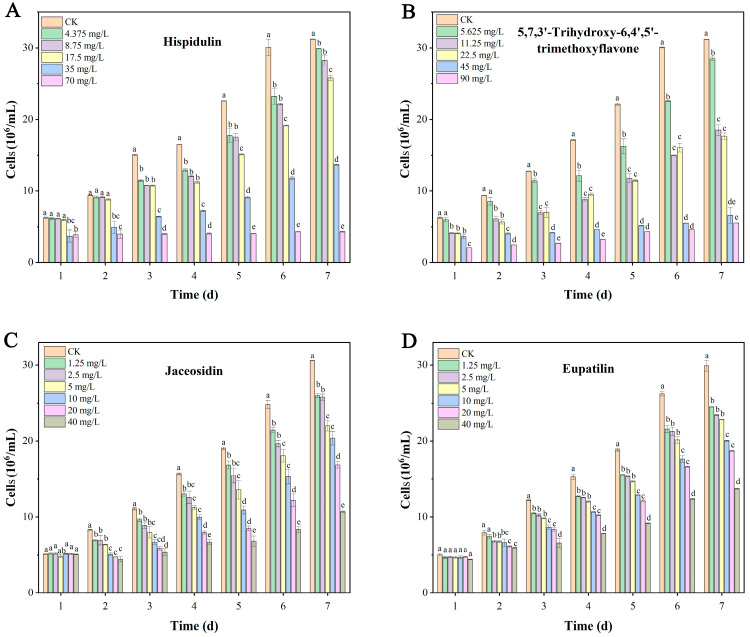
Cell densities of *M. aeruginosa* after treatments with hispidulin (**A**), 5,7,3′-trihydroxy-6,4′,5′-trimethoxyflavone (**B**), jaceosidin (**C**), and eupatilin (**D**). The results presented are the mean of three replicates ± SE; different letters denote significant differences at *p* < 0.05 according to one-way ANOVA with an LSD test.

Six flavonoids and two coumarins present at relatively high levels in DE were selected for evaluation of their anti-algal activities. The results showed that eight compounds exhibited varying degrees of growth inhibition against *M. aeruginosa* over extended exposure times. By day 7, 5,7,3′-trihydroxy-6,4′,5′-trimethoxyflavone, hispidulin, jaceosidin, and eupatilin exhibited IRs exceeding 50%, whereas isofraxidin, chrysosplenetin B, scopoletin, and 7-hydroxycoumarin showed IRs below 50% (Figure S1). Subsequently, concentration-gradient assays were conducted for hispidulin, jaceosidin, 5,7,3′-trihydroxy-6,4′,5′-trimethoxyflavone, and eupatilin. Hispidulin at 35 and 70 mg/L significantly inhibited *M. aeruginosa* cell growth starting from day 1. Significant inhibitory activities were observed for all hispidulin concentrations from days 3 to 7 (Figure 6A). 5,7,3′-Trihydroxy-6,4′,5′-trimethoxyflavone demonstrated significant inhibitory activity throughout the entire treatment period at concentrations ≥11.25 mg/L. Furthermore, significant inhibition was evident even at its lowest tested concentration (5.625 mg/L) starting from day 3 (Figure 6B). No significant difference in algal cell growth was observed between the jaceosidin-treated groups and the control on day 1. However, from day 2 onwards, cell densities under all jaceosidin concentrations were significantly lower than the control (Figure 6C). Eupatilin exhibited a similar pattern to jaceosidin: no significant effect was found on day 1, but concentrations ≥ 2.5 mg/L showed significant inhibitory activity from day 2. Starting on day 3, significant inhibition was observed at all eupatilin concentrations (Figure 6D). Based on the IRs against *M. aeruginosa*, the IC_50_ values on day 7 for hispidulin, jaceosidin, 5,7,3′-trihydroxy-6,4′,5′-trimethoxyflavone, and eupatilin were determined to be 26.23, 27.62, 32.02, and 34.98 mg/L, respectively.

## 4. Discussion

Many species from the Asteraceae family, especially those belonging to the *Artemisia*, *Acmella*, and *Bidens* genera, can influence the development of other species by allelopathy. Secondary metabolites such as flavonoids, terpenes, and alkaloids from the Asteraceae family were considered important allelochemicals with potent phytotoxic action [28,29]. In addition to exhibiting significant inhibitory effects on seed germination and seedling growth of target plants, many *Artemisia* species also demonstrate allelopathic activity against algae. Through co-culture experiments, Xu et al. found that *A. lavandulaefolia* possessed algal-inhibiting effects. Furthermore, organic solvent extracts of *A. lavandulaefolia* significantly suppressed the growth of *M. aeruginosa* [30]. Aqueous extract of *A. herba alba* inhibited the growth of *M. aeruginosa* in a concentration-dependent way. The Chl a and carotenoid contents in the algae cells decreased especially in the 1% treatment group [31]. Among the different organic solvent extracts of *A. annua*, the ethyl acetate extract exhibited the strongest inhibitory effect on *M. aeruginosa* [32]. Furthermore, artemisinin with strong anti-algal activity was identified as the main allelochemical of *A. annua* using an activity-guided fractionation. Artemisinin decreased the soluble protein content and increased the superoxide dismutase (SOD) activity and ascorbic acid content of *M. aeruginosa* but exerted no effect on the soluble sugar content. The results suggested the mode of action of artemisinin on algae may primarily be the increasing level of reactive oxygen species (ROS) in algae cells [33]. The inhibitory effects of *A. lavandulaefolia* on *M. aeruginosa* were tested by co-culturing of plant and algae. The results show that *A. lavandulaefolia* strongly suppressed the cell density of *M. aeruginosa* with an IR of 93.3% on the 10th day of cultivation. Therefore, *A. lavandulaefolia*, which causes the reduction in the habitat’s carrying capacity of algae, may have great potential in controlling algae bloom in eutrophic water [34]. The aqueous extract of *A. argyi* showed inhibitory effects on the growth of *M. aeruginosa*. Additionally, inhibitory action was associated with the oxidative damage and antioxidant reactions of algae cells. Gas chromatography–mass spectrometry (GC-MS) showed that terpenoids have the highest content in the *A. argyi* aqueous extract [25]. These findings collectively demonstrated that species of the *Artemisia* genus possess algal-inhibiting activity. Notably, the aqueous extract of *A. argyi* exhibited significant inhibitory effects against *M. aeruginosa*. This study specifically investigated the anti-algal activity of organic solvent extracts derived from *A. argyi*. The ethanol extract demonstrated potent inhibitory activity, and subsequent bioassay-guided fractionation revealed that among four partitioned fractions, the dichloromethane extract exerted the most potent inhibitory effect on *M. aeruginosa*. This indicates that the dichloromethane fraction contains substantial amounts of algal-inhibiting allelochemicals and represents the primary active fraction.

The mechanisms by which plant allelochemicals inhibit algae are intricately linked to the specific classes of allelochemicals. These allelochemicals primarily suppress algal growth through the inhibition of photosynthesis, disruption of cellular enzyme activity, induction of oxidative damage, and destruction of cell membrane integrity [4]. The growth of algae is dependent on photosynthesis, with Chl a and carotenoids serving as the primary photosynthetic pigments involved in processes such as energy capture and transfer during cell photosynthesis [35]. Consequently, their content can serve as a monitoring indicator for the potential photosynthetic capacity of *M. aeruginosa*. The addition of allelochemicals can disrupt chlorophyll synthesis or promote chlorophyll degradation, leading to a reduced chlorophyll content. PBPs, including PC, APC, and PE, constitute auxiliary light-harvesting/transfer protein complexes essential for fundamental energy conversion in cyanobacteria. Together with conjugated linoleic acids, they absorb most incident solar energy and are crucial for electron/energy transfer and energy production during photosynthesis. In *M. aeruginosa*, PBPs transfer absorbed energy to algal chlorophyll a (i.e., Photosystem II, PSII). These PBPs also represent the primary functional units for photosynthesis in cyanobacteria [36].

Previous studies indicated that plant allelochemicals have the ability to influence photosynthesis and induce oxidative stress in algae cells. Total flavonoids from *Zanthoxylum bungeanum* leaves, which display allelopathic effects on *M. aeruginosa*, could disrupt the structures of oxygen-evolving complexes at the donor side of the PSII reaction center and influence energy distribution at PSII reaction centers, thereby inhibiting electron transmission activities at both the donor and receptor sides of the PSII reaction center [37]. Among several organic solvent extracts of *Landoltia punctata*, the ethyl acetate extract showed the strongest inhibitory effects on *M. aeruginosa* with an IC_50_ of 59.6 mg/L. The contents of Chl a and PBPs of *M. aeruginosa* were decreased under the stress of ethyl acetate extract, indicating that the photosynthesis of *M. aeruginosa* was inhibited. Moreover, the contents of SOD, MDA, and H_2_O_2_ of cell pellets were increased, indicating that the algal cells were damaged by oxidation [38]. Root exudates of *Pistia stratiotes* Linn. reduced the PC content and the PC/APC ratio in the photosynthetic system of *M. aeruginosa*. Meanwhile, the electrical conductivity (EC) and superoxide anion radical (O^2−^) values in the *M. aeruginosa* culture fluid increased, indicating that the allelochemicals released from the root of *P. stratiotes* inhibited algae growth by affecting photosynthesis, destroying the cell membrane, and increasing oxidative damage of *M. aeruginosa* [39]. The filtrate of *Ulva intestinalis* significantly inhibited growth, decreased the Chl a and carotenoid contents, and decreased the maximum PSII quantum efficiency (Fv/Fm) and the effective quantum yield of the PSII photochemistry (*Φ*PSII) values of the bloom-forming cyanobacteria *Nodularia spumigena* and *Nostoc* sp. [40]. The photosynthetic pigments of *M. aeruginosa*, including Chl a, carotenoids, PC, APC, PE, and total PBPs, were decreased after treating with the anti-algal allelochemical gramine. The result indicated that gramine seriously influenced algal photosynthetic activity by destroying the photosynthetic pigments [41]. The expression of *psbD1*, *psaB,* and *rbcL* related to the photosynthesis of *M. aeruginosa* was influenced by the three flavonoids 5,4′-dihydroxyflavone, luteolin, and apigenin [42]. In this study, compared to the control group, prolonged exposure to DE resulted in decreased Chl a and carotenoid contents in *M. aeruginosa* cells. This indicates that DE inhibited photosynthesis by degrading photosynthetic pigments within algal cells. Chl a inhibition exhibited both time- and dose-dependency, with higher DE concentrations demonstrating significantly enhanced algistatic effects. Elevated concentrations of DE correlated strongly with increased photosynthetic inhibition and reduced PBP contents and may impair energy transfer from PBPs to Chl a. Consequently, the light-harvesting capacity may be compromised, ultimately damaging the photosynthetic system. This suppressed synthesis of PC, APC, and PE, demonstrating that *A. argyi* extracts disrupt photosynthetic pigment biosynthesis. The significant pH suppression in the treatments with DE indicated the loss of membrane integrity leading to leakage of acidic intracellular contents (e.g., organic acids, H^+^). The concentration-dependent EC increase in the treated groups revealed reversible changes in membrane permeability with selective leakage of ions (e.g., K^+^, Na^+^). The SEM observations corroborate these findings, providing insights into the anti-algal characteristics of DE: low concentrations primarily disrupt membrane function, while high concentrations induce physical membrane rupture. DE triggers oxidative cascades by inducing ROS bursts (H_2_O_2_), inhibiting CAT activity, and promoting lipid peroxidation (increasing MDA) and protein damage (decreasing TP). The cumulative membrane damage is consistent with lipid peroxidation. The results suggest that the anti-algal mechanisms of DE were probably dependent on the inhibition of photosynthesis, the induction of oxidative damage, and then the destruction of the cell membrane of *M. aeruginosa*.

Flavonoids are a group of natural products with variable phenolic structures. Flavonoids exhibit antioxidant, antibacterial, anti-inflammatory, anti-mutagenic, and anti-carcinogenic activities and are widely used in fields such as food and pharmaceuticals [43]. In addition, in the plants, flavonoids are specifically released into the rhizosphere by roots where they are involved in allelopathy. More and more flavonoids have been characterized in autotoxicity and allelopathic interference [44]. Zhao et al. demonstrated that flavonoids exhibit inhibitory activity against microalgal growth. Quercetin, kaempferol, and luteolin significantly suppress both growth and photosynthetic activity in *M. aeruginosa* cells. At 96 h, these compounds achieved IRs of 83.06%, 81.46%, and 76.47% on algal cell density, and 81.54%, 80.76%, and 80.68% on the Chl a content, respectively [45]. Luteolin from pomegranate peel extract had obvious inhibitory effects on *M. aeruginosa* growth. Moreover, the allelochemical caused inhibition of photosynthesis in *M. aeruginosa* cells by influencing the Chl a content and Fv/Fm [46]. Quercetin significantly inhibited *M. aeruginosa* growth in a concentration-dependent manner. Furthermore, quercetin affects all the photosynthetic fluorescence parameters of algae cells [47]. Kaempferol inhibited the growth of *M. aeruginosa* with an IC_50_ of 3.5 mg/L. Moreover, a mixture of kaempferol and luteolin at an equitoxic ratio exerted additive effects on *M. aeruginosa* growth [48]. The flavonoid 5,4′-dihydroxyflavone showed strong inhibitory effects on microalgae growth [49]. The inhibitory actions of the allelochemical were dependent on inducing ROS and the PCD process [50], downregulating iron/zinc ion transport and toxin synthesis [51], and influencing the photosynthetic activity in *M. aeruginosa* [42,49]. In this study, among the eight most abundant compounds annotated in DE, six were flavonoids. Bio-assays revealed that the flavonoids exhibited different inhibitory activities against *M. aeruginosa*. Among them, hispidulin, jaceosidin, 5,7,3′-trihydroxy-6,4′,5′-trimethoxyflavone, and eupatilin demonstrated strong anti-algal effects. These compounds are likely the main allelochemicals responsible for the anti-algal activity in *A. argyi* leaves.

In addition to flavonoids, there are several known allelochemicals in DE with a relative content of more than 0.5% that exhibit anti-algal activity. For example, the allelochemical salicylic acid inhibited the growth of *Cladophora oligoclona*. The Chl a content of *C. oligoclona* progressively decreased with increasing salicylic acid concentration, demonstrating a dose–response relationship. After 96 h of exposure, a concentration of 0.4 g/L salicylic acid achieved a growth IR of 73.64% against *C. oligoclona*. The 96 h IC_50_ was determined to be 0.262 g/L [52]. Salicylic acid also exhibited inhibitory activity against *M. aeruginosa*. The inhibitory effect became more pronounced with increased allelochemical concentration and prolonged exposure time. At a concentration of 0.12 g/L, salicylic acid achieved an IR of 95.66% against *M. aeruginosa* after 48 h [53]. Additionally, an environmentally friendly sustained-release microsphere of salicylic acid showed long-term inhibition effects on *M. aeruginosa* and could effectively reduce the concentration of MC-LR [54]. The allelochemical α-linolenic acid, which was generally extracted from diverse macroalga, significantly inhibited the growth of *Prorocentrum donghaiense*, a HAB-inducing algae in the marine ecosystem [55]. Azelaic acid from mangrove plant root exudates showed strong allelopathic inhibition on the growth of the marine algae *Prorocentrum micans*, indicating that the allelochemical may play an important regulatory role in mangrove ecosystems [56]. Therefore, the anti-algal activity of DE relies on its diverse allelochemical constituents. Among these, flavonoids exhibit relatively high abundance and potent inhibitory effects, representing the primary allelochemicals. However, other allelochemicals also contribute to the overall effect. The combined action, the selectivities toward different algae species, and the ecological safeties of these allelochemicals need more study.

## 5. Conclusions

This study explored the effects of *A. argyi* extracts on the growth and photosynthesis of *M. aeruginosa* and characterized new anti-algal allelochemicals. EE exhibited strong inhibitory effects on *M. aeruginosa*. Among the extracts obtained using different solvents, DE demonstrated the most significant allelopathic effect and was found to be the primary fraction concentrating the algae-inhibiting active constituents. DE effectively suppressed the synthesis of Chl a, carotenoids, and PBPs, thereby impairing the photosynthetic efficiency of the algal cells. Moreover, DE could induce an overaccumulation of ROS, together with reduced CAT activity and an increased MDA content, indicating lipid peroxidation of the cell membrane. The results were consistent with the changes in EC and pH of the algal culture after DE treatments and were further confirmed by SEM-observed membrane shrinkage in *M. aeruginosa* cells. Consequently, the inhibition of algal cell growth by DE primarily occurs through the disruption of photosynthesis and induction of peroxidation damage. Among the secondary metabolites in DE, four flavonoids with high relative contents, including hispidulin, jaceosidin, 5,7,3′-trihydroxy-6,4′,5′-trimethoxyflavone, and eupatilin, displayed significant algae-inhibiting allelopathic activities, establishing them as the principal anti-algal allelochemicals derived from *A. argyi*. These findings indicated that *A. argyi* possesses remarkable anti-algal activities with extensive application potential in CyanoHAB control, highlighting its commercial viability in environmentally friendly algal control technologies.

## Figures and Tables

**Table 1:** LC-HRMS annotation of main secondary metabolites from DE.

No.	Proposed Compounds	Molecular Formula	MW	Mass Error (ppm)	Main Fragment MS2	RT (min)	Relative Content (%)
1	Esculetin	C_9_H_6_O_4_	178.0264	−1.15	177.0191	23.043	0.623
2	Camphor	C_10_H_16_O	152.1199	−1.17	152.012, 109.1344, 95.2021, 81.0131, 69.0119	24.898	0.937
3	7-Hydroxycoumarin	C_9_H_6_O_3_	162.0315	−1.07	162.0317	25.069	3.474
4	Scopoletin	C_10_H_8_O_4_	192.0421	−1.02	193.0492, 178.0261, 133.0658, 176.9143, 148.0137, 161.0233, 150.0305, 122.035	25.153	5.537
5	Isofraxidin	C_11_H_10_O_5_	222.0525	−1.36	220.8, 191	25.388	2.342
6	Atractylenolide I	C_15_H_18_O_2_	230.1304	−1.27	213.1259, 185.1321, 105.0701, 79.0548	26.228	2.009
7	Azelaic acid	C_9_H_16_O_4_	188.1047	−0.9	187.0974, 142.0039, 99.9256	26.343	0.655
8	Arglabin	C_15_H_18_O_3_	246.1252	−1.57	247.1358	26.773	4.001
9	Salicylic acid	C_7_H_6_O_3_	138.0315	−1.46	93.0344	26.898	2.154
10	Linderane	C_15_H_16_O_4_	260.1045	−1.56	243.1012, 261.1117	27.601	1.822
11	Abscisic acid	C_15_H_20_O_4_	264.1358	−1.45	219.1389, 204.1154	28.272	0.605
12	Dehydrocostus lactone	C_15_H_18_O_2_	230.1304	−1.27	231.1377, 232.1044, 233.1448	28.62	0.719
13	Eupafolin	C_16_H_12_O_7_	316.0579	−1.19	168.0537	28.903	1.377
14	7-Methoxycoumarin	C_10_H_8_O_3_	176.0471	−1.27	145.0287, 135.0442, 117.034	29.638	0.823
15	Linderalactone	C_15_H_16_O_3_	244.1095	−1.76	245.1168	29.659	2.024
16	Hispidulin	C_16_H_12_O_6_	300.0629	−1.59	301.0712, 286.1991, 258.0529, 241.1807, 169.1003, 133.1023, 299.0557	30.941	4.377
17	Iristectorigenin B	C_17_H_14_O_7_	330.0734	−1.72	316.0132	30.95	0.805
18	5,7,3′-Trihydroxy-6,4′,5′-trimethoxyflavone	C_18_H_16_O_8_	360.0839	−1.73	361.0911	31.252	6.754
19	Jaceosidin	C_17_H_14_O_7_	330.0734	−1.76	316.0912, 301.1475	31.423	14.614
20	Eupatilin	C_18_H_16_O_7_	344.0889	−2.06	297.0838, 284.2992	34.216	25.187
21	Chrysosplenetin B	C_19_H_18_O_8_	374.0994	−2	375.1066, 397.0884, 771.1879	35.316	8.442
22	*α*-Linolenic acid	C_18_H_30_O_2_	278.224	−2.22	279.0944, 149.0236, 123.1172, 109.1066, 137.1324, 135.1166, 121.0283	42.933	0.857

## Data Availability

Data are contained within the article and Supplementary Materials.

## Supplementary Materials

The following supporting information can be downloaded at: https://www.mdpi.com/article/10.3390/biology14091141/s1, Table S1: LC-HRMS annotation of secondary metabolites with relative concentrations < 0.5% in DE, and Figure S1: Cell densities of *M. aeruginosa* after treatments with eupatilin, jaceosidin, 5,7,3′-trihydroxy-6,4′,5′-trimethoxyflavone, hispidulin, chrysosplenetin B, isofraxidin, scopoletin, and 7-hydroxycoumarin at 50 mg/L.

## Abbreviations

The following abbreviations are used in this manuscript:

CyanoHABs	harmful cyanobacterial blooms
LC-HRMS	high-resolution mass spectrometry
CE	crude ethanol extract of *A. argyi* leaves
PEE	petroleum ether extract of *A. argyi* leaves
DE	dichloromethane extract of *A. argyi* leaves
EE	ethyl acetate extract of *A. argyi* leaves
WP	water phase of *A. argyi* leaves
IC_50_	half-maximal inhibitory concentration
Chl a	chlorophyll a
PBPs	phycobiliproteins
PCD	programmed cell death
MC-LR	microcystin-leucine-arginine
IR	inhibition rate
PC	phycocyanin
APC	allophycocyanin
PE	phycoerythrin
ROS	reactive oxygen species
SOD	superoxide dismutase
GC-MS	gas chromatography–mass spectrometry
PSII	photosystem II
EC	electrical conductivity
MDA	malondialdehyde
SEM	scanning electron microscopy
CAT	catalase
TP	total protein
