# Supplementary material for: Inhibitory Effects of Artemisia argyi Extracts on Microcystis aeruginosa: Anti-Algal Mechanisms and Main Allelochemicals
Source: Biology (Basel). 2025 Aug 29;14(9):1141. doi: 10.3390/biology14091141 (PMC12467351; doi:10.3390/biology14091141)

Table S1, LC-HRMS annotation of secondary metabolites with relative concentrations < 0.5% in DE.

No.	Proposed Compounds	Molecular Formula	MW	Mass Error (ppm)	Main Fragment MS2	RT (min)	Relative content (%)
1	Quinic acid	$C_7H_{12}O_6$	192.0632	-0.97	191.0559	1.581	0.462
2	2-Pyrrolidinecarboxylic acid	$C_5H_9NO_2$	115.0632	-1.55	70.0651, 116.11	1.592	0.055
3	Stachydrine	$C_7H_{13}NO_2$	143.0945	-1.22	144.1017	1.598	0.182
4	Uridine	$C_9H_{12}N_2O_6$	244.0693	-1.02	245.0431, 110.0246, 189.2	5.829	0.006
5	5-Hydroxymethylfurfural	$C_6H_6O_3$	126.0315	-1.21	109.0283, 127.0388	20.888	0.464
6	Protocatechualdehyde	$C_7H_6O_3$	138.0315	-1.42	119.0148, 109.0301	21.872	0.357
7	Gentisic acid	$C_7H_6O_4$	154.0264	-1.48	153.0192	22.14	0.007
8	Trigonelline HCl	$C_7H_7NO_2$	137.0475	-0.99	137.0445, 136.0616	22.202	0.217
9	Cryptochlorogenic acid	$C_{16}H_{18}O_9$	354.0946	-1.27	193.0494	22.436	0.253
10	Vicenin II	$C_{27}H_{30}O_{15}$	594.1581	-0.55	337.0366, 303.087, 281.1053, 255.0655, 135.008, 119.0504	22.782	0.008
11	1-Caffeoylquinic acid	$C_{16}H_{18}O_9$	354.0947	-1.22	191.056, 179.0345, 135.0449, 193.0494	23.011	0.011
12	Vanillic acid	$C_8H_8O_4$	168.042	-1.49	151.038, 123.41, 65.0386, 151.993	23.081	0.039
13	Homoveratrumic acid	$C_{10}H_{12}O_4$	196.0733	-1.45	197.0806	23.081	0.096
14	4-Methoxyphenylacetic acid	$C_9H_{10}O_3$	166.0628	-1.33	165.056, 164.895	23.112	0.013
15	Schaftoside	$C_{26}H_{28}O_{14}$	564.1473	-1.06	563.1401	23.356	0.094
16	*p*-Hydroxybenzaldehyde	$C_7H_6O_2$	122.0366	-1.72	121.0294, 92.2206	23.463	0.175
17	Fraxetin	$C_{10}H_8O_5$	208.0369	-1.44	208.036	23.756	0.075
18	(*R*)-Mandelic acid	$C_8H_8O_3$	152.0472	-1.19	106.85	23.854	0.032
19	Ligustilide	$C_{12}H_{14}O_2$	190.0991	-1.59	91.0543, 173.0967, 163.1124, 155.0862, 149.0602, 145.1018, 117.0703	23.939	0.221
20	*p*-Coumaric acid	$C_9H_8O_3$	164.0472	-0.86	116.9285, 120.0534, 121.0292	24.503	0.132
21	2-Hydroxy-4-methoxybenzaldehyde	$C_8H_8O_3$	152.0471	-1.47	153.0544	24.514	0.096
22	Hyperoside	$C_{21}H_{20}O_{12}$	464.0951	-0.73	301.034, 151.004,	24.663	0.029

					465.1032, 303.0502, 153.0145, 137.0297		
23	Isochlorogenic acid B	$C_{25}H_{24}O_{12}$	516.1262	-1.08	257.0585, 515.1189	25.07	0.095
24	Pinoresinol 4-*O*-glucoside	$C_{26}H_{32}O_{11}$	520.1937	-1.51	357.1343, 151.0399	25.174	0.013
25	Fraxinellone	$C_{14}H_{16}O_3$	232.1095	-1.72	233.1168	25.179	0.075
26	2-Adamantanone	$C_{10}H_{14}O$	150.1043	-1.03	151.1116	25.374	0.338
27	Xanthoxyline	$C_{10}H_{12}O_4$	196.0733	-1.15	197.0806	25.458	0.032
28	3,5-Dicaffeoylquinic acid	$C_{25}H_{24}O_{12}$	516.1262	-1.06	515.119, 553.0446	25.479	0.300
29	Astragalin	$C_{21}H_{20}O_{11}$	448.1002	-0.8	461.1667, 284.0861, 255.0923, 227.0988	25.488	0.025
30	Isochlorogenic acid C	$C_{25}H_{24}O_{12}$	516.1262	-1.05	257.0558, 515.119	25.894	0.350
31	Perillene	$C_{10}H_{14}O$	150.1043	-1.03	150.1274, 134.096, 105.069, 93.0697, 81.0699, 69.0334	25.961	0.447
32	Germacrone	$C_{15}H_{22}O$	218.1668	-1.23	219.1749	26.538	0.128
33	Curcumenol	$C_{15}H_{22}O_2$	234.1616	-1.64	235.1698	27.457	0.161
34	Linarin	$C_{28}H_{32}O_{14}$	592.1788	-0.71	447.1433, 285.1149, 270.0522, 242.0552	27.802	0.053
35	Coumarin	$C_9H_6O_2$	146.0367	-0.76	103.0122	27.845	0.432
36	Isoalantolactone	$C_{15}H_{20}O_2$	232.1460	-1.36	215.1431, 187.1482, 151.075, 133.0644	28.356	0.357
37	Eriodictyol	$C_{15}H_{12}O_6$	288.0631	-1.15	269.0454, 259.0609, 225.055, 163.0032, 149.0239, 135.0083, 121.0306, 109.0298	28.474	0.209
38	4-Methoxysalicylic acid	$C_8H_8O_4$	168.0420	-1.41	151.038, 123.41, 65.0386	28.597	0.014
39	Luteolin	$C_{15}H_{10}O_6$	286.0474	-1.37	285.0412, 151.004, 241.8977, 269.0451, 153.0178	28.714	0.177
40	Naringenin	$C_{15}H_{12}O_5$	272.0681	-1.56	271.1478, 151.2211, 119.0258	30.552	0.281
41	Apigenin	$C_{15}H_{10}O_5$	270.0524	-1.59	269.2025, 117.112	30.639	0.474
42	Curcumol	$C_{15}H_{24}O_2$	236.1773	-1.25	254.3158, 219.3037	30.804	0.330
43	Hesperetin	$C_{16}H_{14}O_6$	302.0785	-1.71	153.1246	30.872	0.306
44	Parthenolide	$C_{15}H_{20}O_3$	248.1409	-1.49	248.1023	30.887	0.446
45	α-Cyperone	$C_{15}H_{22}O$	218.1668	-1.23	201.1635, 219.174	33.158	0.103
46	Hydroxygenkwanin	$C_{16}H_{12}O_6$	300.0629	-1.56	301.0712, 286.1991, 258.0529, 241.1807, 169.1003, 133.1003	33.222	0.038
47	Artemisinic acid	$C_{15}H_{22}O_2$	234.1616	-1.65	189.11, 199.011	34.898	0.223

48	Chrysin	$C_{15}H_{10}O_4$	254.0575	-1.71	241.2174, 225.0558, 209.0607, 151.0033	35.243	0.074
49	Acacetin	$C_{16}H_{12}O_5$	284.0681	-1.19	285.0736, 270.0534, 242.0585, 153.0187, 133.065	35.498	0.345
50	Pinocembrin	$C_{15}H_{12}O_4$	256.0732	-1.36	255.066	35.676	0.022
51	Phenethyl caffeate	$C_{17}H_{16}O_4$	284.1045	-1.35	135.046	36.074	0.013
52	Pectolinarigenin	$C_{17}H_{14}O_6$	314.0785	-1.74	300.0648, 285.0412, 257.0452	36.13	0.385
53	Glycitein	$C_{16}H_{12}O_5$	284.0681	-1.16	267.9025, 239.9346	36.813	0.023
54	Artemetin	$C_{20}H_{20}O_8$	388.1149	-2.48	389.1222	38.337	0.482
55	Clareolide	$C_{16}H_{26}O_2$	250.1928	-1.87	327.114, 316.9256, 313.2147, 341.3032,	44.529	0.022
56	Isosteviol	$C_{20}H_{30}O_3$	318.2165	-9.48	319.2273, 351.2156	45.381	0.016
57	Linolenic acid ethyl ester	$C_{20}H_{34}O_2$	306.2552	-2.09	307.4802	45.478	0.007
58	Lupenone	$C_{30}H_{48}O$	424.3698	-1.81	407.3665, 425.377	49.723	0.037
59	Vitamin D2	$C_{28}H_{44}O$	396.3385	-1.87	298.2327	53.371	0.006

Supplementary Figure S1. Cell densities of *M. aeruginosa* after treatments with eupatilin, jaceosidin, 5,7,3'-trihydroxy-6,4',5'-trimethoxyflavone, hispidulin, chrysosplenetin B, isofraxidin, scopoletin, and 7-hydroxycoumarin at 50 mg/L.

Commented [Ed1]: Attention AE: Please change “Cell” to “Cells” in the y-axis label.

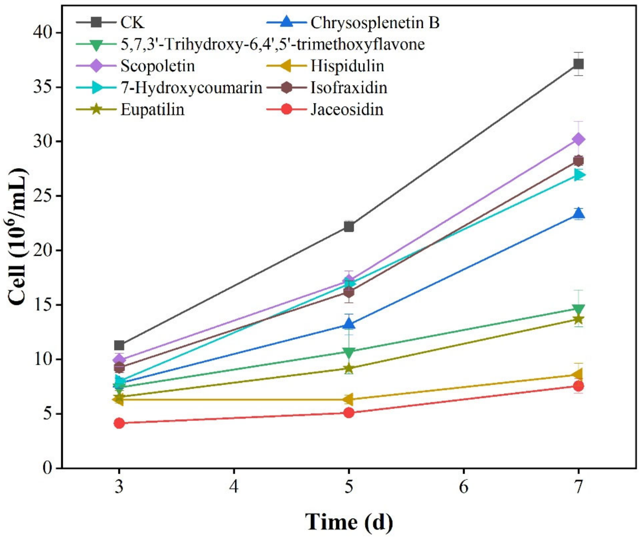

Supplement: Supplementary file 1 [file biology-14-01141-s001.zip › biology-3811518-supplementary.pdf]
